# *Mycobacterium bovis* and *M. caprae* in Bulgaria: insight into transmission and phylogeography gained through whole-genome sequencing

**DOI:** 10.1186/s12917-022-03249-w

**Published:** 2022-04-23

**Authors:** Violeta Valcheva, Claudia Perea, Tanya Savova-Lalkovska, Albena Dimitrova, Lukasz Radulski, Igor Mokrousov, Krustyu Marinov, Hristo Najdenski, Magdalena Bonovska

**Affiliations:** 1 Department of Infectious Microbiology, The Stephan Angeloff Institute of Microbiology, Bulgarian Academy of Sciences, 26, Acad. Georgi Bonchev str., 1113 Sofia, Bulgaria; 2 National Veterinary Services Laboratories, United States Department of Agriculture, Ames, IA USA; 3 National Diagnostic and Research Veterinary Medical Institute “Prof. Dr. G. Pavlov”, Sofia, Bulgaria; 4 National Veterinary Research Institute, Pulawy, Poland; 5 Laboratory of Molecular Epidemiology and Evolutionary Genetics, St. Petersburg Pasteur Institute, St. Petersburg, Russia; 6 Military Medical Academy, Sofia, Bulgaria

**Keywords:** Whole-genome sequencing, *Mycobacterium bovis*, *Mycobacterium caprae*, Cattle, Transmission, Phylogeography, Bulgaria

## Abstract

**Background:**

This study aimed to characterize recent *Mycobacterium bovis*/*M. caprae* isolates from Bulgaria by whole-genome sequencing (WGS) to gain a first insight into their molecular diversity, transmission, and position within the global phylogeography of this important zoonotic species.

**Results:**

The isolates were obtained from cattle in diverse locations of Bulgaria in 2015-2020 and were identified by microbiological and PCR assays. WGS data were used for phylogenetic analysis that also included *M. bovis* global dataset. Thirty-seven *M. bovis/caprae* isolates from Bulgaria were studied and 34 of them were SNP genotyped. The isolates were subdivided into 3 major phylogenetic groups. Type Mbovis-13 (Eu2 complex [western Europe and northern Africa]) included one isolate. Mbovis-37 type included 5 isolates outside of known clonal complexes. The Bulgarian *M. caprae* isolates formed a sub-group within the Mcaprae-27B cluster which also included 22 *M. caprae* isolates from Poland, Spain, Germany, and the Republic of Congo. The Bulgarian *M. caprae* isolates share their latest common ancestors with Spanish isolates. The Mbovis-37 group shares a distant common ancestor (pairwise distance 22-29 SNPs) with an isolate from Poland but was very distant (> 200 SNPs) from the rest of the tree. The Mbovis-13 group shares a common ancestor with two human isolates from Germany. Phylogeographically, both *M. bovis* clades had limited circulation in northeastern Bulgaria while the majority of the studied isolates (*M. caprae*) were from central and western provinces. A phylogenetic network-based analysis demonstrated that 11 Bulgarian isolates were separated by 1 to 6 SNPs within four clusters, mostly forming pairs of isolates.

**Conclusion:**

The obtained WGS analysis positioned the Bulgarian isolates within the global phylogeography of *M. bovis*/*M. caprae*. Hypothetically, the observed phylogenetic diversity may not have resulted from livestock trade routes, but instead may reflect the deeply rooted *M. bovis*/*M. caprae* phylogeography of Europe. A high level of genetic divergence between the majority of the studied isolates suggests limited active transmission of bTB in Bulgaria during the survey period. At the same time, a possibility of the endemic presence of circulating bTB strains in the form of the latent persistent disease cannot be ruled out.

**Supplementary Information:**

The online version contains supplementary material available at 10.1186/s12917-022-03249-w.

## Background

Bovine tuberculosis (bTB) is an important zoonosis with serious implications for livestock farming and public health, with greater distribution in Africa and parts of Asia and America [1–3]. The disease caused by *Mycobacterium bovis* poses a serious risk to agriculture in the affected countries due to the loss of livestock, reduced animal productivity and the commercial value of animal by-products. The socio-economic consequences for farmers are also significant [4, 5]. Due to its zoonotic nature, bovine tuberculosis poses a global public health hazard, especially for breeders and slaughterhouse workers [5–7]. Current bTB control programs are mainly based on testing and slaughter, movement restrictions, and post-mortem inspection measures. These programs and the pasteurization of milk have succeeded in eliminating bTB in humans in most developed countries [5]. The disease remains a problem in developing countries due to closer contact between animals and humans and the use of unpasteurized milk [1, 4].

*M. bovis* has a wide range of hosts in the wildlife, being a permanent reservoir of infection, with serious consequences for livestock in the affected countries [8, 9]. Sources of *M. bovis* infection in cattle can be badgers, feral pigs, deer, and others, that release *M. bovis* into the environment [10, 11]. In Bulgaria, the presence of *M. bovis* in badgers, wild boars, and wild birds has also been demonstrated in the 1990s and early twenty-first century, which confirms a natural reservoir of bTB [12–14]. This creates serious difficulties in controlling and eradicating the disease and contributes to the persistence of infection and the regular spread of the disease among domestic animals [8, 15]. In recent decades, research in this area has not been conducted and the role of wildlife in the disease epidemiology cannot be proven with certainty. National Tuberculosis Eradication Program includes only monitoring of cattle on private and public farms.

Recent studies have proven the existence of significant genomic diversity among *M. bovis* strains isolated from different regions of the world [16]. After sequencing the entire genome of *M. bovis* it became possible to use the information obtained for the correct typing and determination of strain-specific markers [16–18]. Whole-genome sequencing (WGS) is increasingly becoming a preferential approach in diagnosis and epidemiological studies. In some developed countries, in recent years, WGS has been used in official bTB control programs, in decision-making on treatment, outbreak response, and surveillance to address the transmission of bovine tuberculosis in farms and/or in wildlife within proven specific outbreaks or countries [19].

*M. tuberculosis* complex is a clonal genetic species with a hierarchical population structure whereas its genome variation is mainly based on SNPs and large genomic deletions. With regard to *M. bovis*, its population structure was previously shown to be shaped by several expansions of the limited number of clones [20]. Spoligotype- and deletion-based classification permitted to delineate four such clonal complexes named European 1, European 2, African 1, and African 2 derived from the ancestral BCG-like spoligotype SB0120 (according to the Mbovis.org nomenclature). Occasional (and not so rare) homoplasy observed in the spoligotype evolution along with low discriminatory power of the method [16] makes spoligotyping definitely less suitable for epidemiological studies of bTB. In contrast, multiple genome-wide SNPs located both in the coding sequences and intergenic regions have negligibly low reversion rate and homoplasy indexes. Accordingly, SNPs present a robust and highly-discriminatory approach to molecular epidemiology, phylogenetics, and population-based studies of *M. tuberculosis* complex, including *M. bovis* [21, 22].

Our previous molecular study for the differentiation of *M. bovis* isolates in Bulgaria was based on spoligotyping [23]. In the present study, we aimed to use WGS to characterize the *M. bovis* /*M. caprae* isolates obtained from different regions of the country in 2015-2020. We sought to gain a first informed view on molecular diversity and transmission as well as phylogeography of the Bulgarian *M. bovis/M. caprae* isolates placed in the global phylogenetic framework of this important zoonotic species.

## Results and discussion

The Program for Monitoring and Control of Bovine Tuberculosis in Bulgaria includes annual, one-time intradermal tuberculin testing with bovine PPD tuberculin of all cattle over 42 days of age. Doubtfully PPD tuberculin-reacted animals undergo differential tuberculin testing with bovine and avian PPD tuberculin on day 42 after the first tuberculin test. Animals positively reacted to regular and differential tuberculinization are sent for sanitary slaughter to isolation slaughterhouses. In herds with detected tuberculosis, all cattle over 42 days of age are tuberculinized every 2 months until negative results are obtained in the intradermal and laboratory tests. The animals are tested again after 6 months and in case of repeated negative results, the herd is considered free of tuberculosis. In 2015-2020, 66 TB-affected farms with 851 TB-positive animals were identified in 11 provinces across the country. Lymph nodes from slaughtered cattle were sent to the National Reference Laboratory for bacteriological and PCR тesting and confirmation of results. All 851 cases were confirmed by both PCR and bacteriology, as requested by National bTB Control Program implemented since 2015.

### Position of Bulgarian *M. bovis*/*M. caprae* within global WGS-based phylogeny

The present research included a representative sample of 40 *M. bovis*/*M. caprae* isolates from lymph nodes of TB-positive cattle available in the strain collection in National Diagnostic and Research Veterinary Medical Institute “Prof. Dr. G. Pavlov”, Sofia, Bulgaria. These isolates represent the areas with the most affected farms and those with isolated cases of the disease that represent 60.6% of the infected herds in 2015-2020.

Of the 40 DNA samples from bacterial isolates, 37 were found of sufficient quality and concentration to be suitable for WGS. Of them, 3 isolates had low-quality sequencing reads and were excluded from further analysis and 34 isolates were successfully SNP genotyped (Additional file 1 with Table S1). Phylogenetic analysis was carried out on the 34 Bulgarian isolates along with a selection of genomes from a global dataset of the *M. bovis* WGS data compiled and curated at the National Veterinary Services Laboratories, United States Department of Agriculture, Ames, IA, USA, including publicly available genomes in the NCBI Sequence Read Archive (Additional file 2 with Table S2). High-quality SNPs were identified that clustered the isolates into smaller more manageable groups. Groups were created based on the number of isolates as well as the evolutionary distance. We identified the most recent common ancestor (MRCA) in the groups. Then, the most closely related isolate was selected based on having the fewest number of SNPs differences, and a pairwise comparison of SNPs to the MRCA was recorded.

In addition, we performed in silico spoligotyping of the analyzed strains and assignment of the international code number according to Mbovis.org database (Table S1). In total, three spoligotypes were identified and included 28 *M. caprae* isolates (SB0418), 5 *M. bovis* isolates (SB0120) and 1 *M. bovis* isolate (SB0339). Some of the studied isolates were previously subjected to classical macroarray-based spoligotyping [23]. Both in silico and experimental spoligotypes were available for 24 isolates and showed 100% concordance.

**Fig. 1:**
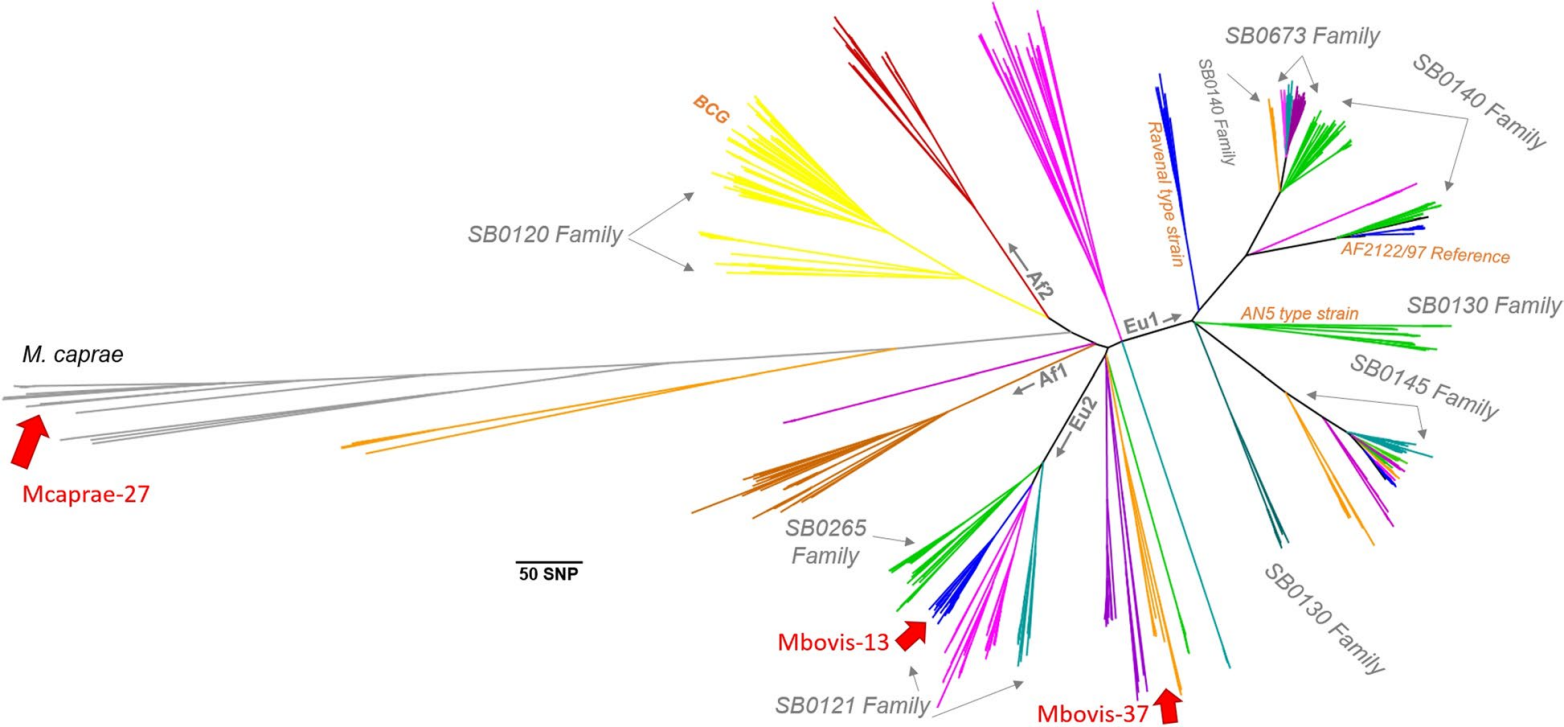
Low-resolution phylogenetic tree of *M. bovis*/*M. caprae* isolates. The isolates from Bulgaria fall into three major clades which are indicated by the red arrows

**Fig. 2:**
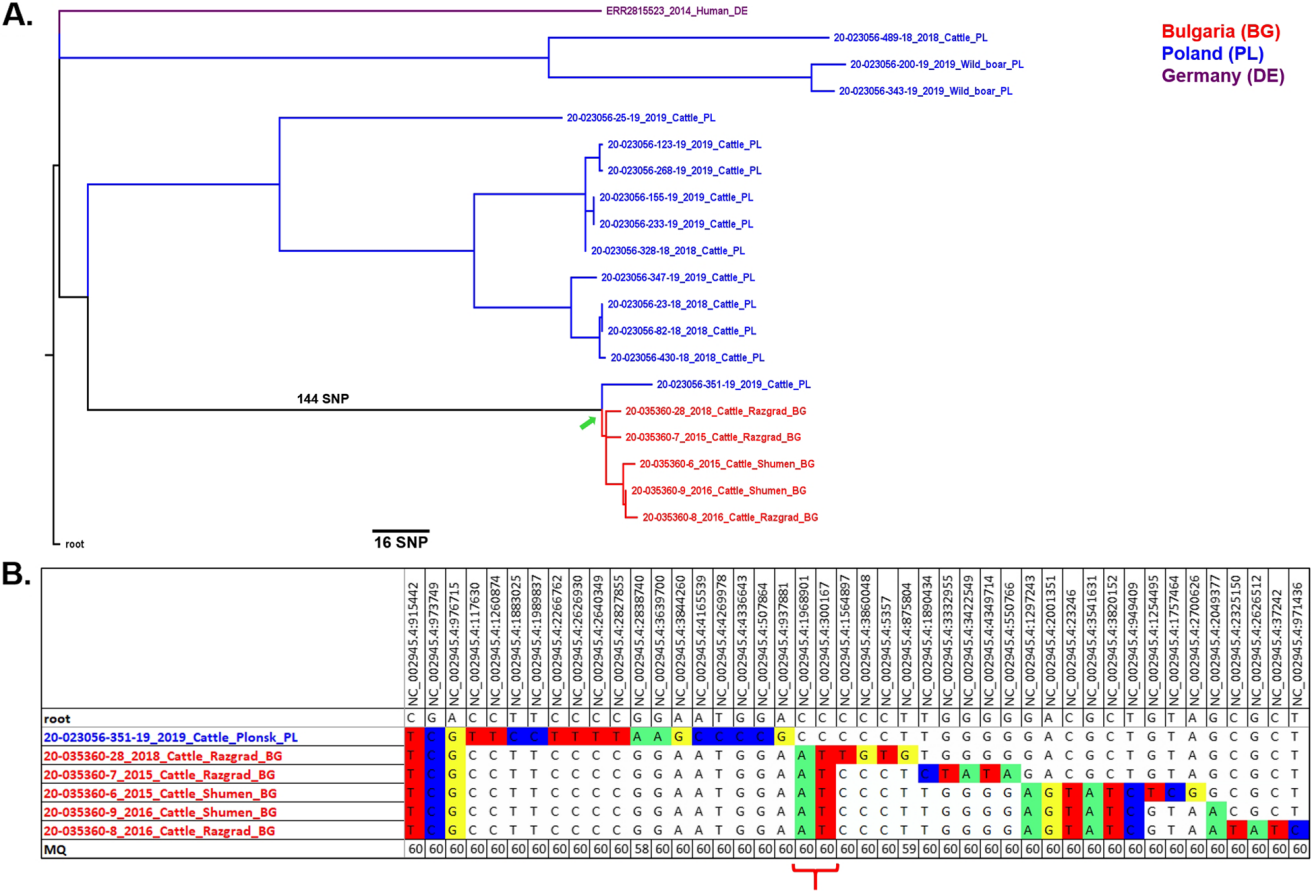
**A** High-resolution phylogenetic tree of Mbovis-37 isolates. The isolates from Bulgaria are only 2 SNPs from sharing a common ancestor with an isolate from Poland (green arrow). **B** Abbreviated SNP table of Mbovis-37 isolates from Bulgaria. Red bracket represents the common ancestor to the five Bulgarian isolates

The Bulgarian *M. bovis*/*M. caprae* isolates fall into 3 major phylogenetic groups identified in this work as Mbovis-13, Mbovis-37 and Mcaprae-27B (Fig. 1). The groups were different in size and phylogenetic relationships. The Mbovis-37 group included 5 isolates from Bulgaria. These isolates share a distant common ancestor with isolates from Poland (Fig. 2A). The most recent common ancestor to these isolates has only accumulated 2 SNP (red bracket) since sharing a common ancestor with the isolate from Poland (green arrow). Furthermore, the Mbovis-37 isolates from Bulgaria are very closely related, all being ≤11 SNP from a shared common ancestor (Fig. 2B). They differ by 22-29 SNPs from their closest known relative (023056-351-19) an isolate from Poland but are distinct from all other isolates by (> 200) SNPs. Mbovis-37 group does not belong to any of the clonal complexes currently defined for *M. bovis* (Eu1, Eu2, Af1, Af2, and BCG-like).

**Fig. 3:**
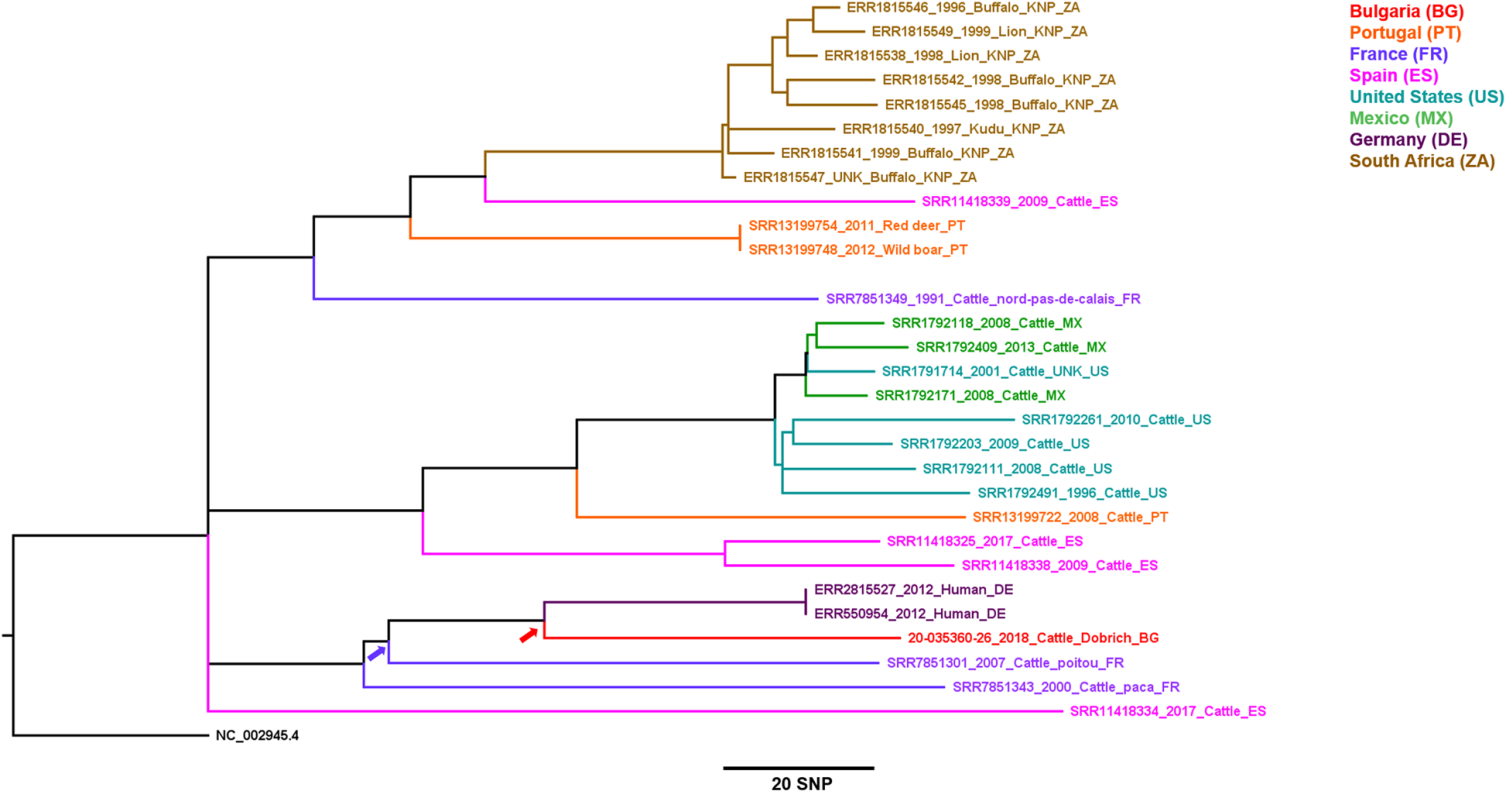
High-resolution phylogenetic tree of Mbovis-13 isolate. The red arrow represents the common ancestor shared by an isolate from Bulgaria and two isolates from Germany. The purple arrow represents the common ancestor shared by these with isolates from France

Only one Bulgarian isolate belongs to the Mbovis-13 group (Fig. 1). Based on the global SNP framework, this group corresponds to the European Complex 2, thought to have evolved on the Iberian Peninsula and distributed throughout western continental Europe and some regions of northern Africa [18, 24, 25]. The Mbovis-13 isolate shares its most recent common ancestor with two isolates from humans in Germany (red arrow); altogether this German/Bulgarian group shares a common ancestor with an isolate from France (purple arrow) (Fig. 3).

**Fig. 4:**
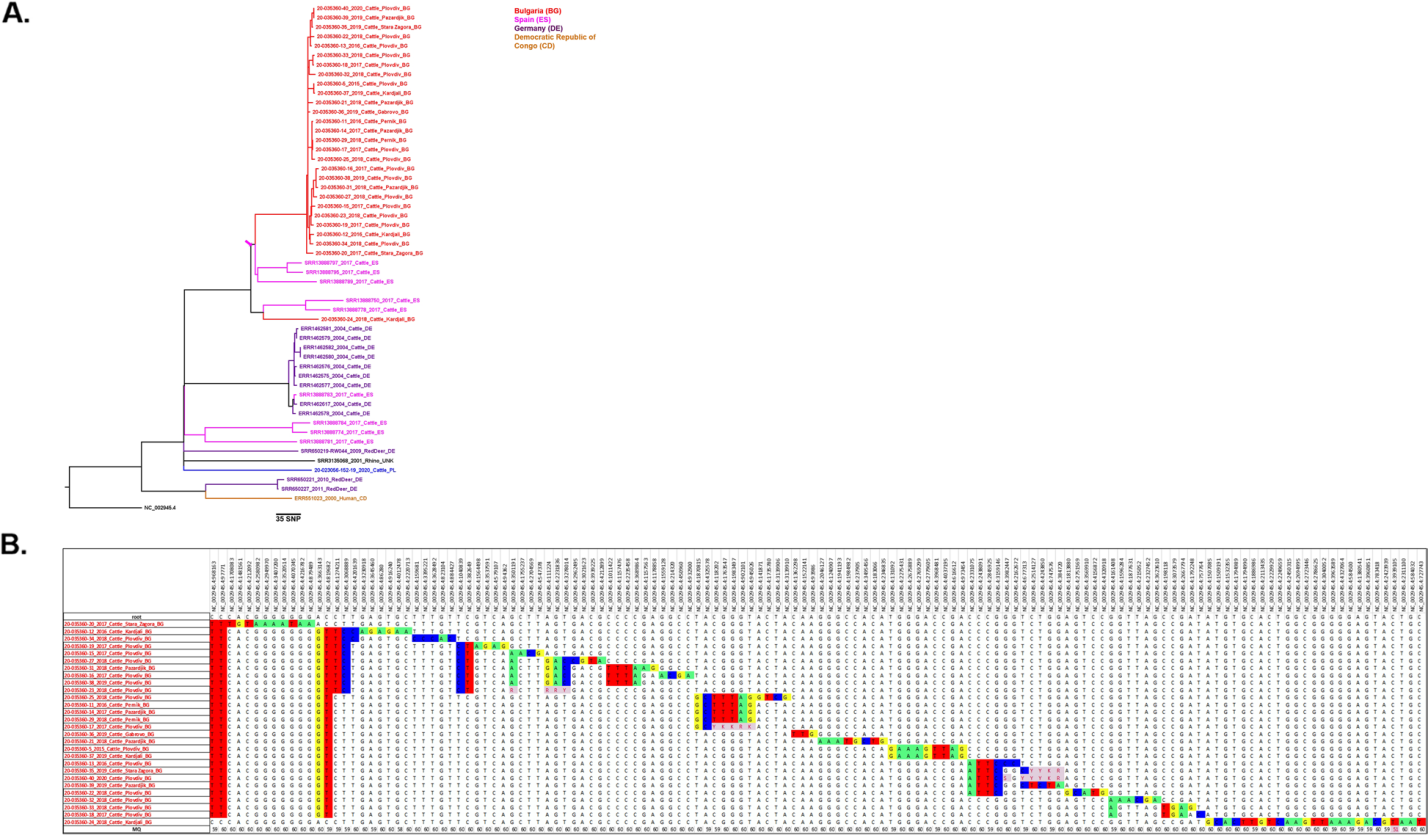
**A** High-resolution phylogenetic tree of Mcaprae-27B isolates from Bulgaria. **B** Abbreviated SNP table for Mcaprae-27B isolates from Bulgaria. The pink arrow represents the most recent common ancestor to these isolates

**Fig. 5:**
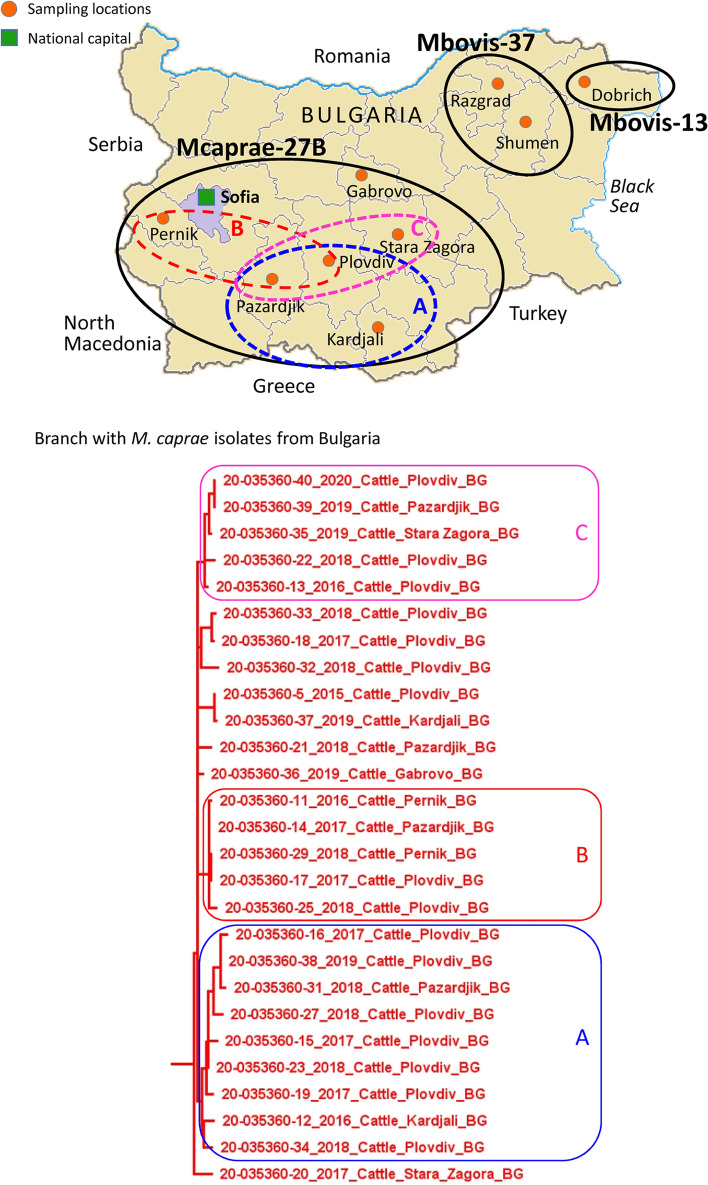
Geographic distribution of the main *M. bovis*/*M. caprae* phylogenetic groups identified by WGS in Bulgaria

Finally, the largest group of 28 *M. caprae* isolates clustered in the Mcaprae-27B branch (Fig. 4). This branch includes isolates from Poland, Spain, Germany, the Republic of Congo and one of unknown origin (labeled as Rhine in Fig. 4). The isolates from Bulgaria share the most recent common ancestors with isolates from Spain (pink arrows). Almost all the *M. caprae* isolates from Bulgaria form a unique clade of closely genetically related isolates, sharing a common ancestor within ≤15 SNP. Three distinct subclusters were observed within this branch that we have labeled as A, B, and C for convenience of discussion of their geographic specificity at the intra-country level (Fig. 5).

We additionally compared our WGS results to the recently proposed new classifications to define *M. bovis* phylogenetic lineages and sublineages [18, 26, 27]. Zwyer et al. [27] proposed to divide *M. bovis*, *M. caprae* and *M. orygis* in three main phylogenetic lineages named La1 (further subdivided into La1.1-La1.8), La2, and La3. We assigned the Bulgarian clusters to these lineages as follows: Mbovis-37 to La1.7; Mcaprae-27B to La2; Mbovis13 to La1.7.1. Zimpel et al. [18] proposed four distinct global lineages of *M. bovis* labeled as Lb (Lb1, Lb2, Lb3, and Lb4). Based on their classification, Bulgarian isolates of the Mbovis-13 and Mbovis-37 clusters were assigned to Lb3.

A subset of sequences representing each of the clonal complexes described by Loiseau et al. [26] was retrieved from NCBI and a phylogenetic analysis was performed adding the Bulgarian isolates to determine their location in the phylogeny. The Bulgarian sequences fell in European Complex 2, other unknowns, and *M. caprae*. Reference genomes *M. bovis* AF2122/97, *M. bovis* BCG, *M. bovis* AN5, and *M. bovis* Ravenal were also included for additional perspective (Additional file 3 with Fig. S1).

The geographic diversity of the studied isolates is illustrated in the map in Fig. 5A. Our previous study provided the first insight into phylogeography of *M. bovis/caprae* in Bulgaria based on spoligotyping. In spite of the well-known limitation of that method, a certain gradient was observed at the within-country level. Classical *M. bovis* types predominated in the Northeast, while *M. caprae* was prevalent in Central and Southwestern Bulgaria. By spoligotyping, these *M. caprae* isolates belonged mostly to the Central/Eastern European cluster [23].

In the present study, higher resolution WGS/SNP typing confirmed these findings. The identified *M. bovis* isolates were from Northeastern Bulgaria. The five isolates from the Mbovis37 group (Fig. 2A) did not belong to any well-defined cluster and were found in the districts of Razgrad and Shumen. The most genetically distinct isolate from the Mbovis13 group (Fig. 3) was from the Dobrich district.

**Fig. 6:**
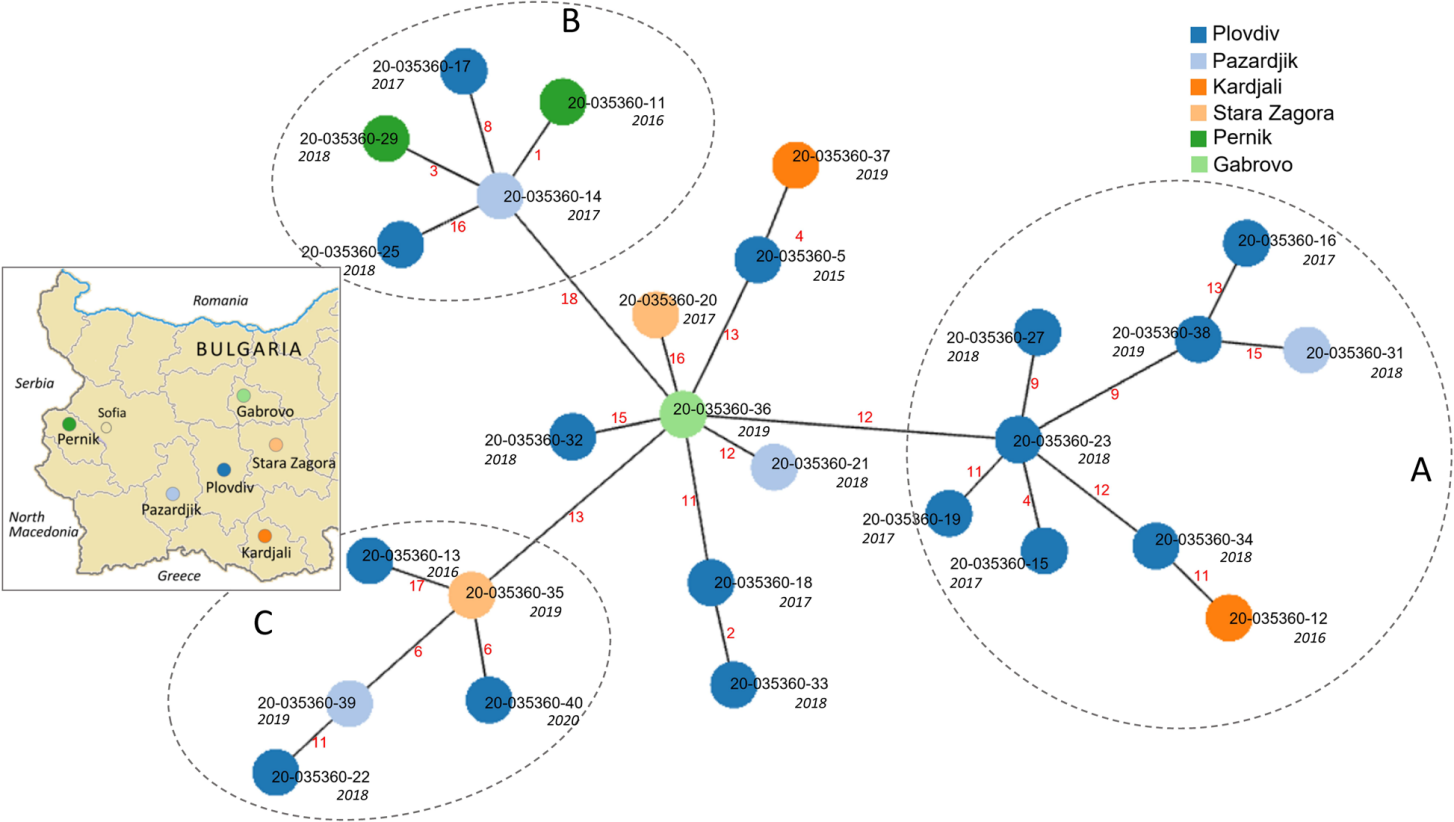
Minimum spanning tree of the Mcaprae-27B isolates from Bulgaria. The same color is used to designate the location on the tree and the inserted map. The year of isolation is shown below isolate ID. The number of SNPs between isolates is shown in red on the connecting branches

The most numerous sample of isolates belong to the Mcaprae-27B group (Fig. 4A) and its three subclusters A, B and C predominate in the central and southwestern part of the country (Fig. 5). *M. caprae* isolates of all three subclusters have been identified in the districts of Pazardjik and Plovdiv. In addition, subcluster A includes an isolate from Kardjali, subcluster B – two isolates from Pernik, and subcluster C – an isolate from Stara Zagora district (Fig. 5). These three subclusters are also clearly distinguished on the minimum spanning tree of the Mcaprae-27B group (Fig. 6).

The identified phylogenetic relationships of the studied isolates from cattle in Bulgaria and other countries observed in Figs. 2 and 3 may be partly explained by livestock trade contacts with regard to livestock commercial chains. Concerning this particular study, data on the live cattle export from and import to Bulgaria from available reports are helpful but changing trade directions through years should be taken into consideration. For example, the recent annual report on livestock 2018-2019 in Bulgaria [28] mentioned the Czech Republic, Germany, and Hungary as sources of live cattle imports to Bulgaria, but not Spain nor Poland.

On the other hand, the very long branch (144 SNPs) that separates Bulgarian Mbovis-37 isolates from most of those from Poland, poses a different scenario (Fig. 2a). Given such a large genetic distance, it is difficult to decipher the source of these isolates.

The Polish isolate 20-023056-351-19_2019_cattle_PL was the closest relative to Bulgarian Mbovis-37 with the distance of 22-29 SNPs. This strain came from Płońsk county in the Masovian Voivodeship in central Poland and it was the only isolate from this location in the national database. Unfortunately, the epizootic investigation failed to identify the source of the infection. This individual in the past was in several different farms where tuberculosis was not recorded. However, there are suspicions that the infection occurred in the Płock county, where the tested cow lived in 2014. In 2014, many bTB cases were recorded in this county. There is a high probability that *M. bovis* transmission may have occurred between neighboring herds. A distance of 22-29 SNPs separating this Polish isolate from the closest Bulgarian isolate seems too long to speculate about recent links. However, when this distance is translated into > 30 years to their divergence this is reminiscent about the time when two countries were economically united within the Soviet bloc. While the well-known Warsaw Treaty was a military union, there was also The Council for Mutual Economic Assistance focused on economic development and exchange that existed from 1949 to 1991 (https://en.wikipedia.org/wiki/Comecon).

The Bulgarian *M. caprae* are also quite distant from their closest known relatives from Spain in the Mcaprae-27B group, with at least a 150 SNP genetic distance (Fig. 4A). Similar to the above reasoning, they are distantly related by WGS analysis. It is possible that more closely related intermediate strains may be extinct because of slaughter.

### Genetic diversity of Bulgarian *M. caprae*/*M. bovis* and insight into transmission

Previously, it was suggested for human *M. tuberculosis* studies that epidemiologically linked isolates consistent with recent transmission differ by five or fewer SNPs. In contrast, isolates differing by more than 12 SNPs are not directly linked, while pairs differing by six to 12 SNPs are indeterminate [29]. The same study calculated the mutation rate within *M. tuberculosis* genome to vary from 0.3 to 0.7 SNP/genome/year while 0.5 is generally accepted as a mean value. Previous *M. bovis* studies estimated mutation rate in different settings and with wildlife hosts from 0.2 SNP/genome/year (95% HPD: 0.1 – 0.3) [30], to 0.37 (95% HPD: 0.24–0.51) [31] and 0.53 (95% HPD: 0.22-0.94) [32]. In view of these heterogeneous estimations, we believe that the mean 0.5 mutation rate used in our study is acceptable.

**Fig. 7:**
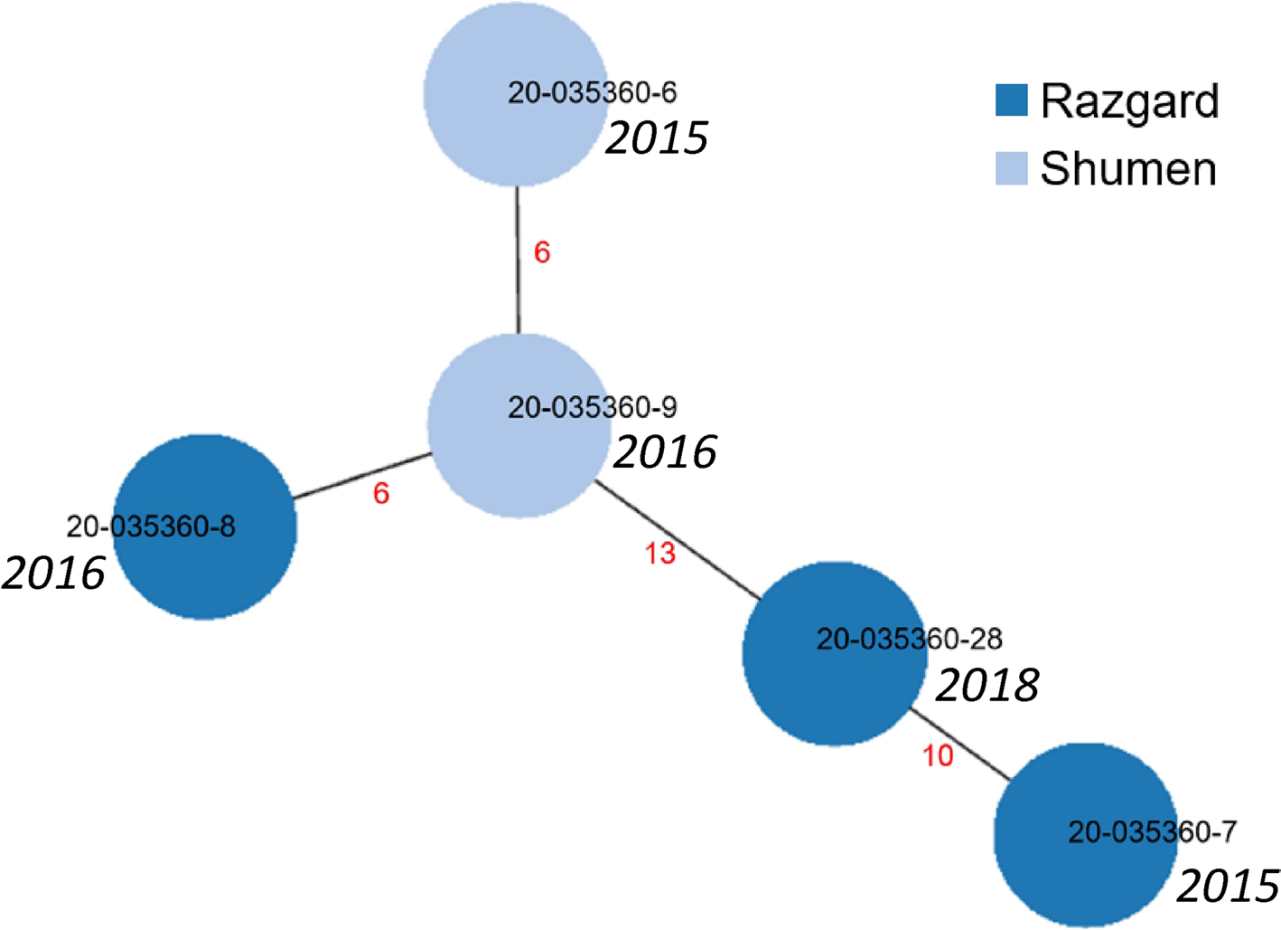
Minimum spanning tree of the Mbovis-37 isolates from Bulgaria. The year of isolation is in italic. The number of SNPs between isolates is shown in red on the connecting branches

In view of these estimations, we interpreted minimum spanning trees (MST) built for two groups of the Bulgarian isolates, Mcaprae-27B (28 isolates, Fig. 6) and Mbovis-37 (5 isolates, Fig. 7). Taken together, 12 Mcaprae-27B and 3 Mbovis-37 isolates were separated by 1 to 6 SNPs within four clusters that could correlate with the relatively recent transmission. Other isolates were more divergent and could not be assigned to the shared clusters.

The MST of the largest group *M. caprae*-27B shows a high diversity (Fig. 6). Three isolates with subcluster B (upper part of the tree) differ by 1-3 SNPs: two isolates from Pernik and one from Pazardjik, both locations are neighboring compared to other locations in this study. Also, in the central part of the tree, we observe two isolates that differ by 4 SNPs, from neighboring provinces of Plovdiv (parental isolate, 2015) and Kardjali (distal derived isolate, 2019). These two isolates are separated by 4 years and their location on the tree (the most recent isolate being the distal one) and their genetic distance of 4 SNPs roughly correlate with the expected mutation rate of 0.3-0.7 SNPs/genome/year. Interestingly, all three *M. caprae* isolates from Kardjali were very genetically distant from one another. The same is true for isolates from Stara Zagora. This may be due to the undersampling of these locations, however, even the largest sample of isolates from Plovdiv showed significant genetic divergence.

The MST tree of the 5 *M. bovis* isolates (Mbovis-37 group) revealed one isolate in the central position and two of the derived isolates differing from it in 6 SNP (Fig. 7). This may indicate a recent transmission however these isolates were recovered in 2015-2016 and no further isolates were identified. Two other *M. bovis* isolates from this group differed from the central node by 10 or more SNPs and were therefore not likely to have undergone recent transmission.

A closer look at the year of isolation reveals that in some instances more distal and derived isolates were isolated earlier than preceding “parental” isolates (Figs. 6 and 7). This discrepancy may suggest that true parental isolates were not effectively isolated because the infected animals were not identified. The discrepancy could not be due to the under-testing because all herds are regularly tuberculin-tested in Bulgaria. Another plausible explanation for this could be that these animals had a latent form of bTB that was not detected during surveillance [33, 34]. The role of wildlife in the transmission of bTB in Bulgaria should be also taken into consideration given the published reports about *M. bovis* confirmed in badger and wild pigs [12–14]. However, these reports date back to 1995-2003, and no papers on bTB in wildlife in Bulgaria were published recently.

Interestingly, *M. caprae* isolates in this study were all from the southern and southwestern parts of Bulgaria. *M. caprae* was initially isolated from goats and is noteworthy that sizeable goat populations are present in the neighboring region of Northern Greece. While data on *M. caprae* zoonotic TB in Greece is limited, dairy goat farm outbreaks and goat to human transmission in northern Greece have been documented since 2005 ([35] and references therein). In Bulgaria, goat breeding is not much developed. Usually, private farmers keep from one to several animals, which are not subject to monitoring and control for the presence of tuberculosis. The above recent study in Northern Greece described *M. caprae* transmission from goat to the goat breeder [35]. The MIRU-VNTR phylogenetic analysis together with reference isolates from MIRU-VNTRplus.org placed that Greek isolate within the branch dominated by SB0418 spoligotype. Noteworthy, all but one *M. caprae* isolates in this Bulgarian study also presented this spoligotype. In this view, a link between neighboring areas of northern Greece and southwestern Bulgaria appears plausible but should be addressed by WGS analysis.

## Conclusions

In conclusion, this WGS survey of *M. bovis*/*M. caprae* isolates from cattle in Bulgaria defined their phylogeographic structure. *M. caprae* was more prevalent in Bulgaria than *M. bovis*. Bulgarian isolates clustered within global clades, but are distinct from isolates from other European countries. Hypothetically, the observed phylogenetic diversity may not only be due to transmission of pathogenic mycobacteria within the livestock trade but may also be the result of the prolonged circulation of such strains in wildlife reservoirs and contact between wildlife and domestic cattle.

The high level of genetic divergence between the majority of the studied isolates suggests a limited active transmission of bTB in Bulgaria during the survey period (lack of clusters beyond a few pairs of related isolates). However, the possiblity of the endemic presence of circulating bTB strains in the form of the latent persistent disease cannot be ruled out. Consequently, this implies the importance of both implementation of WGS and proactive search of bTB in animals and meat.

The obtained WGS data on the specificity and geographical diversity of the studied *M. bovis*/*M. сaprae* isolates from cattle in Bulgaria provides a basis for a broader and more in-depth epidemiological study of circulating strains in the country and transmission of tuberculosis in cattle. The inclusion of the whole genome sequencing in the official program for control and eradication of bovine tuberculosis in the country will update and improve diagnostic approaches, provide information that will increase success and efficiency in decision-making to reduce the transmission of infection among cattle.

## Methods

### Study collection and strain identification

Diagnostic materials from lymph nodes of slaughtered animals that responded positively or doubtfully to tuberculin test were received from different regions of Bulgaria and tested in the National Reference Laboratory for tuberculosis in animals (Sofia, Bulgaria) according to the ongoing bTB control program [36]. Following macroscopic (pathoanatomical) observation for the presence of tuberculous lesions, tissue suspensions of all samples were prepared for microbiological and PCR assays in accordance with World Organization for Animal Health protocols [3].

The samples were homogenized and decontaminated with BBL MycoPrep™ Specimen Digestion/Decontamination Kit (BD, USA), containing N-acetyl-L-cysteine and sodium hydroxide (NALC-NaOH). The sediment obtained was resuspended in 1 mL of phosphate buffer. Further, each sample was inoculated in parallel on liquid and solid media: 500 μL was cultured in MGIT tubes, 100 μL on Löwenstein-Jensen medium with pyruvate and 100 μL in Stonebrink medium with pyruvate and PACT at 37 °C and 5% CO2. The primary growth in the liquid medium was observed after 5-10 days, and on solid media after 3-8 weeks. The smears prepared from the cultures were stained with Ziehl-Neelsen kit (BD TB ZN warm staining kit; Liofilchem, Italy) to detect the presence of acid-fast bacteria.

DNA was extracted directly from tissue samples with the NucleoSpin® Tissue kit (Macherey-Nagel GmbH & Co. KG) and cultured mycobacterial strains with the Seeplex MTB/NTM ACE kit (Seegene, USA). *M. bovis* was identified using the *Mycobacterium bovis* amplification kit (Genekam Biotechnology AG, Germany). The isolates were previously tested by spoligotyping followed by comparison with SITVIT_WEB (http://www.pasteur-guadeloupe.fr:8081/SITVIT2/). This permitted to assign them to *M. bovis* and *M. caprae* [23].

### WGS and phylogenetic analysis

WGS and data analysis were performed at the United States Department of Agriculture (USDA), National Veterinary Services Laboratories, USA (NVSL). The isolates were sequenced on a MiSeq instrument (Illumina, San Diego, CA, United States) using 2 × 250 paired-end chemistry and the Nextera XT library preparation kit (Illumina, San Diego, CA, United States). Multiple isolates were indexed per lane, providing approximately 50-100X coverage per isolate. Raw FASTQ files were analyzed with the vSNP pipeline (https://github.com/USDA-VS/vSNP), which includes quality processing as first step. Reads were aligned to reference genome *M. bovis* AF2122/97 (NCBI RefSeq Accession NC_002945.4) using BWA-mem [37]. SNPs were called using FreeBayes [38] and visually validated with IGV [39]. Phylogenetic trees were constructed based on whole genome concatenated SNP sequences using RAxML [40] under a GTR-CAT model of substitution. Tree visualization, annotation, and editing were performed with FigTree [41]. As an output from the vSNP pipeline, SNP tables for each phylogenetic group were generated; these were formatted Excel tables that group and sort isolates and SNPs with respect to their phylogenetic pattern. In the SNP tables, the columns identify the genome location of the SNP calls and the isolates are listed in the rows. The reference call is listed across the top and the SNPs are highlighted. Map-quality for each SNP is indicated on the bottom row, which is the average map quality of all isolates at that position (60 is the highest possible score). Phylogenetic groups were identified as previously [42]. *M. bovis* genomes available from NCBI SRA and the USDA database were included in the phylogenetic analysis along with Bulgarian strains.

Minimum spanning trees were obtained with PHYLOViZ software [43] using the concatenated SNP alignments for each group (in fasta format) as input.

The in silico spoligotyping of the WGS data was performed using SpoTyping program (https://github.com/xiaeryu/SpoTyping-v2.0) and the profiles were assigned to the international codes by comparison with Mbovis.org database (www.mbovis.org).

## Supplementary Information

**Additional file 1: Supplementary Table 1.** Sequencing statistics of Bulgarian isolates.

**Additional file 2: Supplementary Table S2.** Publicly available genomes included for global analysis.

**Additional file 3: Figure S1.** Phylogenetic tree of Bulgarian isolates with world isolates. Based on classification by Loiseau et al., 2020 [26].

## Data Availability

WGS data of the Bulgarian *M. bovis*/*M.caprae* isolates were deposited at the NCBI Sequence Read Archive (accession number: PRJNA785819). A complete list of publicly available genomes included for the global analysis is provided in Supplementary Table S2.

## Abbreviations

bTB: Bovine tuberculosis
MRCA: Most recent common ancestor
SNP: Single nucleotide polymorphism
SRA: Sequence read archive
WGS: Whole-genome sequencing

## References

[1] Ayele WY, Neill SD, Zinsstag J, Weiss MG, Pavlik I (2004). Bovine tuberculosis: an old disease but a new threat to Africa. Int J Tuberc Lung Dis.

[2] World Health Organization. The challenges of preventing bovine tuberculosis. Bull World Health Organ. 2018;96:82–3. 10.2471/BLT.18.020218. 10.2471/BLT.18.020218 PMC5791786 29403109

[3] OIE (2018). Terrestrial manual, bovine tuberculosis, Ch. 3.4.6.

[4] Cosivi O, Grange JM, Daborn CJ, Raviglione MC, Fujikura T, Cousins D, Robinson RA, Huchzermeyer HF, de Kantor I, Meslin FX (1998). Zoonotic tuberculosis due to *Mycobacterium bovis* in developing countries. Emerg Infect Dis.

[5] Michel AL, Muller B, van Helden PD (2010). *Mycobacterium bovis* at the animal-human interface: a problem, or not?. Vet Microbiol.

[6] Olea-Popelka F, Muwonge A, Perera A, Dean AS, Mumford E, Erlacher-Vindel E, Forcella S, Silk BJ, Ditiu L, El Idrissi A (2017). Zoonotic tuberculosis in human beings caused by *Mycobacterium bovis*—a call for action. Lancet Infect Dis.

[7] World Health Organization (WHO) (2019). Global tuberculosis report 2019.

[8] Palmer MV, Thacker TC, Waters WR, Gortázar C, Corner LA (2012). *Mycobacterium bovis*: a model pathogen at the interface of livestock, wildlife, and humans. Vet Med Int Art.

[9] Palmer MV (2013). *Mycobacterium bovis*: characteristics of wildlife reservoir hosts. Transbound Emerg Dis.

[10] Barasona JA, Torres MJ, Aznar J, Gortázar C, Vicente J (2015). DNA detection reveals *mycobacterium tuberculosis* complex shedding routes in its wildlife reservoir the Eurasian wild boar. Transbound Emerg Dis.

[11] Gormley E, Corner LAL (2018). Pathogenesis of *Mycobacterium bovis* infection: the badger model as a paradigm for understanding tuberculosis in animals. Front Vet Sci.

[12] Bachvarova J, Tsvetkov J (1995). The badger host. Tuberculosis among domestic and wild animals is gaining momentum. Hunt Fish.

[13] Bachvarova J, Likov B, Kandov P, Todorov T, Gyurov B, Naidenov V, Tsvetkov J, Nedkova L, Parvulov B, Baichev J, Karov R (1997). Distribution of tuberculosis in game - mammals and birds. Vet Med.

[14] Popova T. Mycobacterium bovis in badger. In: Proceedings of International Scientific Conference "50 years University of Forestry". Sofia: LTU; 2003:102–3.

[15] Buddle BM, Skinner MA, Chambers MA (2000). Immunological approaches to the control of tuberculosis in wildlife reservoirs. Vet Immunol Immunopathol.

[16] Lasserre M, Fresia P, Greif G, Iraola G, Castro-Ramos M, Juambeltz A, Nuñez Á, Naya H, Robello C, Berná L (2018). Whole genome sequencing of the monomorphic pathogen *Mycobacterium bovis* reveals local differentiation of cattle clinical isolates. BMC Genomics.

[17] Garnier T, Eiglmeier K, Camus J-C, Medina N, Mansoor H, Pryor M, Duthoy S, Grondin S, Lacroix C, Monsempe C, Simon S, Harris B, Atkin R, Doggett J, Mayes R, Keating L, Wheeler PR, Parkhill J, Barrell BG, Cole ST, Gordon SV, Hewinson RG (2003). The complete genome sequence of *Mycobacterium bovis*. Proc Natl Acad Sci U S A.

[18] Zimpel CK, Patané JS, Guedes AC, de Souza RF, Silva-Pereira TT, Camargo NC, de Souza Filho AF, Ikuta CY, Neto JS, Setubal JC, Heinemann MB (2020). Global distribution and evolution of *Mycobacterium bovis* lineages. Front Microbiol.

[19] Guimaraes AMS, Zimpel CK (2020). *Mycobacterium bovis*: from genotyping to genome sequencing. Microorganisms.

[20] Smith NH, Dale J, Inwald J, Palmer S, Gordon SV, Hewinson RG, Smith JM (2003). The population structure of *Mycobacterium bovis* in Great Britain: clonal expansion. Proc Natl Acad Sci U S A.

[21] Hauer A, Michelet L, Cochard T, Branger M, Nunez J, Boschiroli ML, Biet F (2019). Accurate phylogenetic relationships among *Mycobacterium bovis* strains circulating in France based on whole genome sequencing and single nucleotide polymorphism analysis. Front Microbiol.

[22] Tazerart F, Saad J, Sahraoui N, Yal D, Niar A, Drancourt M (2021). Whole genome sequence analysis of *Mycobacterium bovis* cattle isolates, Algeria. Pathogens.

[23] Valcheva V, Savova-Lalkovska T, Vyazovaya A, Dimitrova A, Bonovska M, Najdenski H (2020). First insight into phylogeography of *Mycobacterium bovis* and *M. caprae* from cattle in Bulgaria. Infect Genet Evol.

[24] Rodriguez-Campos S, Schürch AC, Dale J, Lohan AJ, Cunha MV, Botelho A (2012). European 2 – a clonal complex of *Mycobacterium bovis* dominant in the Iberian Peninsula. Infect Genet Evol.

[25] Zimpel CK, Brandão PE, de Souza Filho AF, de Souza RF, Ikuta CY, Soares Ferreira Neto J, Soler Camargo NC, Heinemann MB, Guimarães AMS (2017). Complete genome sequencing of *Mycobacterium bovis* SP38 and comparative genomics of *M. bovis* and *M. tuberculosis* strains. Front Microbiol.

[26] Loiseau C, Menardo F, Aseffa A, Hailu E, Gumi B, Ameni G, Berg S, Rigouts L, Robbe-Austerman S, Zinsstag J, Gagneux S, Brites D (2020). An African origin for *Mycobacterium bovis*. Evol Med Public Health.

[27] Zwyer M, Çavusoglu C, Ghielmetti G, Pacciarini ML, Scaltriti E, van Soolingen D, Dötsch A, Reinhard M, Gagneux S, Brites D. A new nomenclature for the livestock-associated *Mycobacterium tuberculosis* complex based on phylogenomics [version 2; peer review: 2 approved]. https://open-research-europe.ec.europa.eu/articles/1-100/v2. Accessed 20 Apr 2022. 10.12688/openreseurope.14029.2 PMC10445919 37645186

[28] Bulgaria (2019). Livestock and products annual report.

[29] Walker TM, Kohl TA, Omar SV, Hedge J, Del Ojo Elias C, Bradley P, Iqbal Z, Feuerriegel S, Niehaus KE, Wilson DJ, Clifton DA, Kapatai G, Ip CLC, Bowden R, Drobniewski FA, Allix-Béguec C, Gaudin C, Parkhill J, Diel R, Supply P, Crook DW, Smith EG, Walker AS, Ismail N, Niemann S, Peto TEA, Modernizing Medical Microbiology (MMM) Informatics Group (2015). Whole-genome sequencing for prediction of mycobacterium tuberculosis drug susceptibility and resistance: a retrospective cohort study. Lancet Infect Dis.

[30] Trewby H, Wright D, Breadon EL, Lycett SJ, Mallon TR, McCormick C, Johnson P, Orton RJ, Allen AR, Galbraith J, Herzyk P, Skuce RA, Biek R, Kao RR (2016). Use of bacterial whole-genome sequencing to investigate local persistence and spread in bovine tuberculosis. Epidemics.

[31] Salvador LCM, O'Brien DJ, Cosgrove MK, Stuber TP, Schooley AM, Crispell J, Church SV, Gröhn YT, Robbe-Austerman S, Kao RR (2019). Disease management at the wildlife-livestock interface: using whole-genome sequencing to study the role of elk in Mycobacterium bovis transmission in Michigan, USA. Mol Ecol.

[32] Crispell J, Zadoks RN, Harris SR, Paterson B, Collins DM, de-Lisle GW, Livingstone P, Neill MA, Biek R, Lycett SJ, Kao RR, Price-Carter M (2017). Using whole genome sequencing to investigate transmission in a multi-host system: bovine tuberculosis in New Zealand. BMC Genomics.

[33] Álvarez AH, Estrada-Chávez C, Flores-Valdez MA (2009). Molecular findings and approaches spotlighting Mycobacterium bovis persistence in cattle. Vet Res.

[34] Lahuerta-Marin A, Milne MG, McNair J, Skuce RA, McBride SH, Menzies FD, McDowell SJW, Byrne AW, Handel IG, de C Bronsvoort BM. (2018). Bayesian latent class estimation of sensitivity and specificity parameters of diagnostic tests for bovine tuberculosis in chronically infected herds in Northern Ireland. Vet J.

[35] Papaventsis D, Dougas G, Kalkouni O, Karabela S, Manika K (2021). Occupational exposure to zoonotic tuberculosis caused by Mycobacterium caprae, northern Greece, 2019. Emerg Infect Dis.

[36] Savova-Lalkovska T, Bonovska M, Dimitrova A, Valcheva V, Petkov Y, Hadjieva G, Najdenski H (2019). Evaluation of classical and rapid methods for isolation and identification of *Mycobacterium bovis* in cattle in Bulgaria. Bulg J Vet Med.

[37] Li H, Durbin R (2009). Fast and accurate short read alignment with burrows-Wheeler transform. Bioinformatics.

[38] Garrison E, Marth G (2012). Haplotype-based variant detection from short-read sequencing.

[39] Robinson JT, Thorvaldsdóttir H, Winckler W, Guttman M, Lander ES, Getz G, Mesirov JP (2011). Integrative genomics viewer. Nat Biotechnol.

[40] Stamatakis A (2014). RAxML version 8: a tool for phylogenetic analysis and post-analysis of large phylogenies. Bioinformatics.

[41] Rambaut A (2009). FigTree, version 1.4.3. Computer program distributed by the author.

[42] Orloski K, Robbe-Austerman S, Stuber T, Hench B, Schoenbau M (2018). Whole genome sequencing of *Mycobacterium bovis* isolated from livestock in the United States, 1989–2018. Front Vet Sci.

[43] Nascimento M, Sousa A, Ramirez M, Francisco AP, Carriço JA, Vaz C (2017). PHYLOViZ 2.0: providing scalable data integration and visualization for multiple phylogenetic inference methods. Bioinformatics.

