# Supplementary material for: Mycobacterium bovis and M. caprae in Bulgaria: insight into transmission and phylogeography gained through whole-genome sequencing
Source: BMC Vet Res. 2022 Apr 23;18:148. doi: 10.1186/s12917-022-03249-w (PMC9034630; doi:10.1186/s12917-022-03249-w)

Figure S1. Phylogenetic tree of Bulgarian isolates with world isolates.
Based on classification by Loiseau et al., 2020.

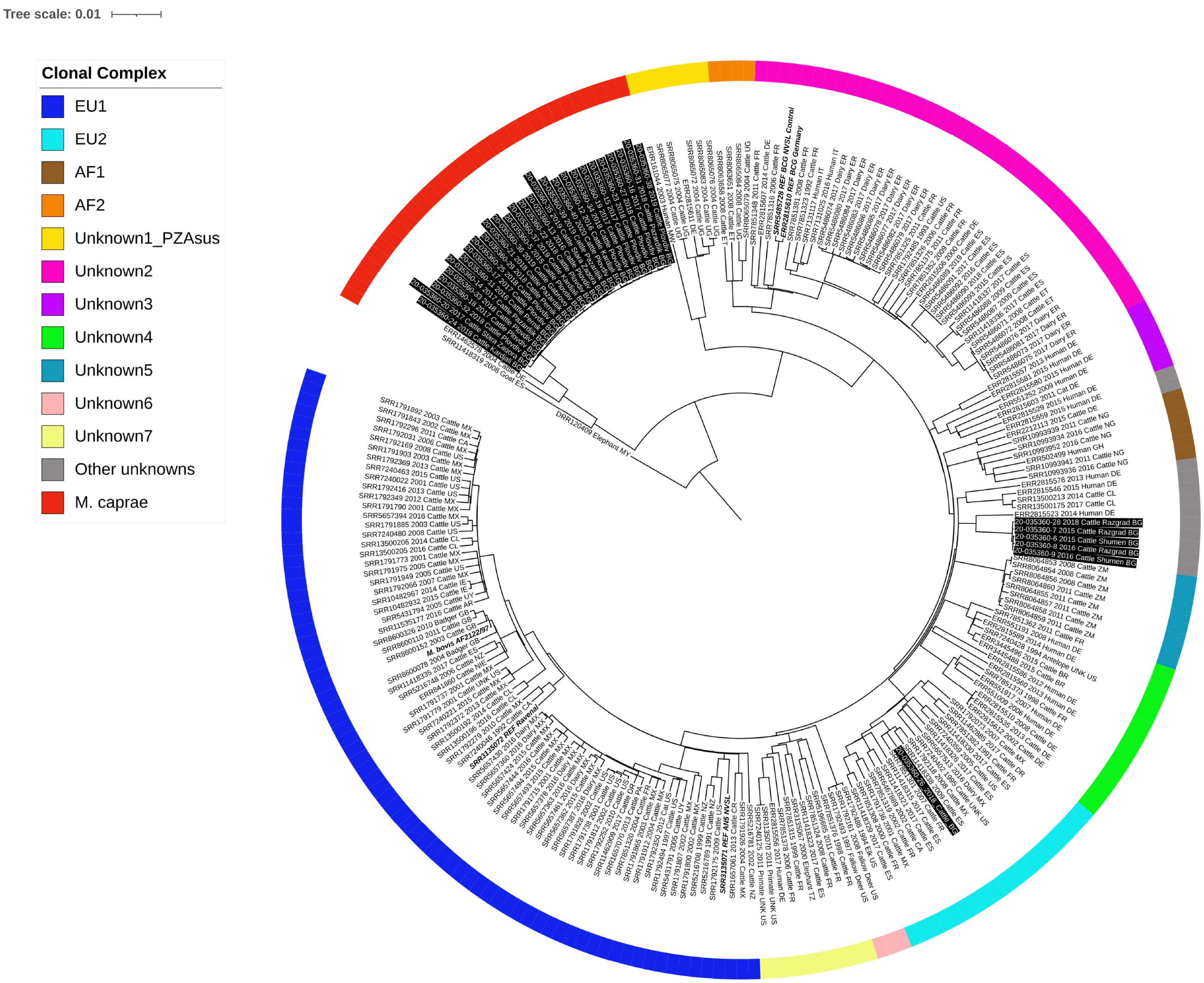

Supplement: Supplementary file 3 — Additional file 3: Figure S1. Phylogenetic tree of Bulgarian isolates with world isolates. Based on classification by Loiseau et al., 2020 [26]. [file 12917_2022_3249_MOESM3_ESM.pdf]
